# Accelerated Senescence and Apoptosis in the Rat Liver during the Progression of Diabetic Complications

**DOI:** 10.21315/mjms2022.29.6.5

**Published:** 2022-12-22

**Authors:** Ratih Yuniartha, Nur Arfian, Wiwit Ananda Wahyu Setyaningsih, Sagita Mega Sekar Kencana, Dwi Cahyani Ratna Sari

**Affiliations:** Department of Anatomy, Faculty of Medicine, Public Health and Nursing, Universitas Gadjah Mada, Yogyakarta, Indonesia

**Keywords:** hyperglycaemia, oxidative stress, apoptosis, cellular senescence, diabetic liver injury

## Abstract

**Background:**

Chronic hyperglycaemia of diabetes causes long-term damage and impaired function of multiple organs. However, the pathological changes in the liver following long-term diabetes remain unclear. This study aimed to determine the pathological complications of long-term diabetes in the rat liver.

**Methods:**

Intraperitoneal injection of streptozotocin (STZ) was used to induce diabetes in rats at a single dose (60 mg/kg body weight [BW]). Rats were euthanised at 1 month (DM1 group), 2 months (DM2 group) and 4 months (DM4 group) following diabetes induction with six rats in each group. Immunohistochemistry was performed against SOD1, CD68, p53 and p16 antibodies. Messenger RNA (mRNA) expressions of SOD1, SOD2, GPx, CD68, p53, p21 and caspase-3 genes were measured by reverse transcription-polymerase chain reaction.

**Results:**

Hepatic p53 mRNA expression was significantly higher in DM1, DM2 and DM4 groups compared to the control group. The p21 and caspase-3 mRNA expressions were significantly upregulated in the DM2 and DM4 groups. The p16-positive cells were obviously increased, particularly in the DM4 group. Bivariate correlation analysis showed mRNA expressions of p21 and caspase-3 genes were positively correlated with the p53 gene.

**Conclusion:**

Diabetic rats exhibited increased apoptosis and senescence in the liver following a longer period of hyperglycaemia.

## Introduction

Diabetes mellitus (DM) is a chronic metabolic disease due to absolute or relative insulin deficiency or insulin resistance which is characterised by glucose intolerance and persistent hyperglycaemia (1). Recent data showed that 537 million adults worldwide suffered from diabetes in 2021 and this number is expected to rise to 643 million in 2030 (2). Diabetes is categorised into type I DM and type II DM, hyperglycaemia in pregnancy and other types of diabetes (2). Type I DM is caused by autoimmune beta-cell destruction in the pancreas, leading to insulin deficiency and the incidence of this type is highest in young adults and children (1, 2). In type II DM, there is an abnormally increased resistance to the action of insulin and insulin production fails to overcome the resistance. Type II DM is the major type of diabetes (> 90% of total diabetes prevalence) and commonly affects older adults and people with overweight or obesity (1, 2).

Chronic hyperglycaemia induces glucose toxicity resulting in irreversible cell damage and prolonged hyperglycaemia leads to the onset of DM (3). Diabetes has serious impacts on its sufferers due to the long-term complications such as, retinopathy, abnormal wound healing, cardiovascular diseases and nephropathy. Chronic hyperglycaemia causes toxicity to various biomolecules (proteins, RNA and DNA) of organelles and cells resulting in organ damage. The generation of reactive oxygen species (ROS) and oxidative stress are associated with the onset of DM and the development of its complications such as diabetic nephropathy and cardiomyopathy (3–6). High blood glucose stimulates the free radical formation by increasing ROS production. The low immune system response in the condition of DM results in the body’s inability to cope with the increased ROS formation causing disruption in the balance between ROS and the antioxidant enzymes that leads to the dominance of oxidative stress conditions (4, 7). DM causes oxidative stress, which adversely affects cellular physiology, including the islets of Langerhans, which have the lowest level of intrinsic antioxidant defenses (7). Hyperglycaemia induces mitochondrial ROS generation through several pathways, including xanthine oxidase, nicotinamide adenine dinucleotide phosphate (NADPH) oxidase, uncoupling nitric oxide synthase, activation of the polyol pathway flux, activation of protein kinase-C (PK-C) and increased advanced glycation end products (AGEs) formation (3, 6, 8).

Prolonged exposure to high glucose levels causes macrovascular and microvascular complications of diabetes in the majority of patients with type I DM and type II DM (4, 9). The detrimental effects of chronic hyperglycaemia and oxidative stress on tissues, including vascular, retina and kidney tissue involve numerous mechanisms or pathways. Several human and animal studies have demonstrated that chronic hyperglycaemia of DM is correlated with the liver morphological changes and the development of liver disease in either type II DM or type I DM (10–12). However, the mechanisms for the pathogenesis remain unclear. Accumulating evidence indicates that oxidative stress and inflammation have a significant role in the development of liver damage in diabetic conditions (13).

It has been reported that hyperglycaemia is closely related to the development of long-term complications of diabetes, such as diabetic nephropathy and cardiomyopathy, in all types of diabetes (14, 15). Several complications of long-term hyperglycaemia are partially due to apoptotic cell death and premature cellular senescence/aging resulting in a decreased organ function. A number of studies have shown that hyperglycaemia directly stimulated apoptosis of the cells in the myocardium, endothelial cells, Schwann cells, podocytes and tubular cells (15–19). Thus, apoptosis plays a role in the development of several diabetic complications such as retinopathy, cardiomyopathy, nephropathy and peripheral neuropathy of diabetes (15–19). In addition, the biochemical environment of diabetic hyperglycaemia induces endogenous stress that accelerates cellular senescence. Cellular senescence is a state of an irreversible arrest of cell proliferation followed by functional and morphological alterations, including increased expression of senescence markers such as the negative regulatory of cell cycle genes and senescence-associated β-galactosidase (SA-β-Gal) (14). It is a main biological mechanism underlying cell aging. A previous study showed that senescence of beta cells plays a crucial role in the development of DM (20). In addition, cellular aging is also associated with the development of diabetes complications. Cellular senescence or premature aging occurs in cells when cultured under hyperglycemic stress and in several conditions, including chronic diabetic ulcers, diabetic cardiomyopathy and nephropathy (14, 15, 21, 22).

The liver is the main metabolic organ that regulates blood level of glucose homeostasis by balancing glycogen breakdown, glucose production and its utilisation. Because of its important role in metabolism, further metabolic disturbances may occur, if the liver is exposed to prolonged hyperglycaemia. Whether hyperglycaemia induces hepatic cell apoptosis and premature senescence in diabetic liver has not been fully clarified. The objective of our study was to determine the effects of hyperglycaemia of diabetes on the liver during the progression from a healthy state to the long-term state of diabetes. The long-term effects of hyperglycaemia were experimentally investigated by evaluating the expression of apoptotic and cellular senescence markers in the livers of type I diabetic rats.

## Materials and Methods

### Animal Model of Diabetes Mellitus

Male Sprague Dawley rats (3 months–4 months) were kept under controlled environmental conditions with lighting (12 h–12 h). A single dose of streptozotocin (STZ) (60 mg/kg body weight (BW); Nacalai Tesque Inc., code 32238–91) was intraperitoneally injected into rats. The level of blood glucose was quantified at day 5 post-injection of STZ to determine the success of the model; the rats with the level greater than 200 mg/dL were considered diabetic. Rats with DM were divided based on duration of diabetes from the point of injection to the termination of the experiment, as follows: 1 months (DM1), 2 months (DM2) and 4 months (DM4) groups, with six rats in each group. The control group was intraperitoneally injected with a single dose of NaCl 0.9%, then euthanised after 4 months. Rats were euthanised under the injection of ketamine anesthesia (60 mg/kg BW–100 mg/kg BW) intramuscularly. Liver tissues were harvested for further analysis, with one part fixed in normal buffer formalin for paraffin embedment and the other part was stored in RNA preservation solution for RNA isolation.

### RNA Isolation, cDNA Synthesis and Reverse Transcription-Polymerase Chain Reaction

The RNA from liver tissues were isolated using Genezol reagent (GENEzol™, Cat No. GZR100) based on the manufacturer’s instructions. RNA concentrations were quantified using the nanodrop spectrophotometer. The synthesis of cDNA was performed using ReverTra Ace (Toyobo, Cat. No. TRT-101), deoxyribonucleotide triphosphate (dNTP) (Takara, Cat. No. 4030) and random primer (Takara, Cat No. 3801) based on the manufacturer’s instructions. The sequence of the primers of the tested genes are listed in Table 1. The reverse transcription-polymerase chain reaction (RT-PCR) was done using Taq Master Mix (GoTaq Green Master Mix, Cat No. M7122). PCR products and DNA ladder (Bioron, Germany, Cat No. 306009) were loaded on 2% agarose gel electrophoresis. The messenger RNA (mRNA) expression was quantified by densitometric analysis using ImageJ software and the mRNA expression of ß actin was used to normalise expression.

### Histological and Immunohistochemical Staining of SOD1, CD68, p53 and p16 from Liver Tissue

The paraffin sections were stained with haematoxylin and eosin. For immunohistochemistry, paraffin sections were deparaffinised in xylene, rehydrated through graded alcohols to water, then heated in a citrate buffer. The sections were treated with 3% H_2_O_2_ in PBS for 30 min to block endogenous peroxidase, then treated with blocking serum. The sections were incubated with primary antibodies at 4 °C overnight, then incubated with species-specific secondary antibodies for 1 h at room temperature. The sections were washed with a wash buffer, incubated with 0.05% 3,3’-diaminobenzidine (DAB) and counterstained with haematoxylin. The primary antibodies included rabbit monoclonal antibody superoxide dismutase-1 (SOD1) (Bioss, bs-10216R; 1:100), mouse polyclonal antibody anti-CD68 (Abcam, ab955; 1:300), rabbit monoclonal antibody p53 (Abcam, ab131442; 1:100) and mouse monoclonal antibody p16INKa (Invitrogen MA5-17142; 1:300).

## Statistical Analysis

The data were statistically analysed using the SPSS Statistics version 23.0 software (IBM Corp., Armonk, NY). Statistical analysis was done using one-way ANOVA followed by a post-hoc LSD test. The normality of the data was analysed using the Shapiro-Wilk test. The correlation among variables was analysed using bivariate Pearson’s correlation and the data were presented as Pearson’s correlation coefficients (*r*-value). The difference of the data was considered statistically significant if the *P*-value was < 0.05.

## Results

### Long-Term Diabetes Caused Morphological Alterations, Decreased Antioxidant Enzymes and Induced Inflammation in the Rat Liver

**Figure 1:**
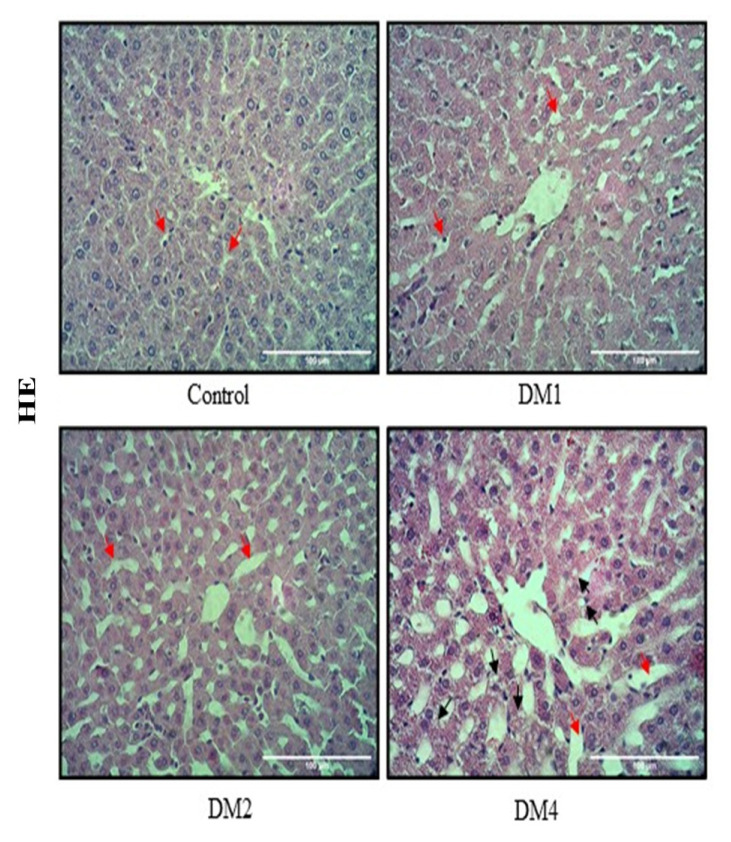
Histological structure of the rat liver. Tissues stained with haematoxylin and eosin (HE). Control rats showing characteristics of normal hepatocytes and normal sinusoids (red arrows), diabetic groups euthanised at 1, 2 and 4 months showing progressive enlargement of sinusoids (red arrows), respectively. Microvesicular steatosis was clearly observed in DM4 group (black arrows)

**Figure 2:**
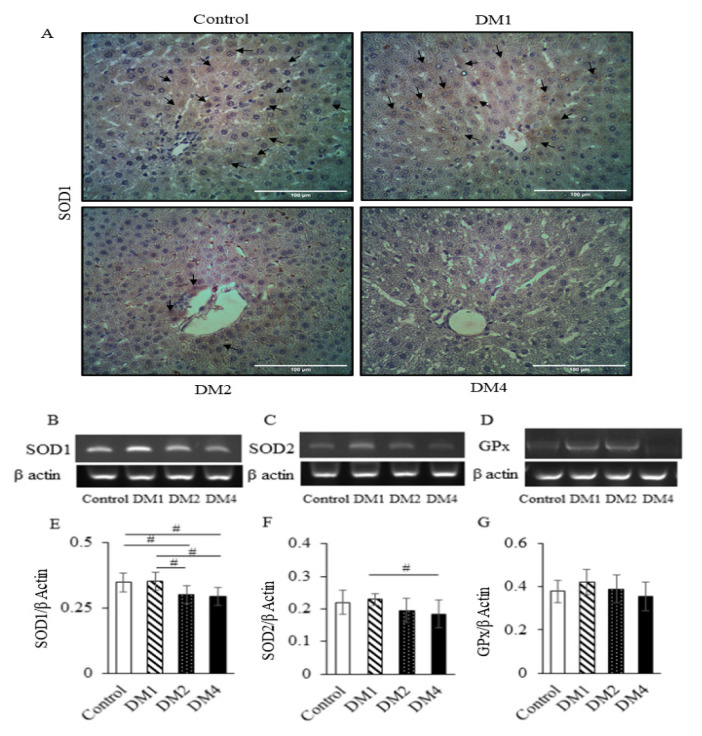
Immunohistochemical staining for SOD1 and mRNA expression of antioxidant enzymes in the rat liver. (A) Hepatic SOD1-positive cell expression. The SOD1 immunostaining of liver cells in the DM1 group showed a stronger brown intensity than the control group. The SOD1-positive cells were progressively decreased in the livers of the DM2 and DM4 groups. Black arrows indicate SOD1-positive cells. (B–D) Representative images of gel electrophoresis from RT-PCR products of SOD1, SOD2 and GPx and its corresponding β actin, respectively. (E–G) Relative quantification of the mRNA expressions of SOD1, SOD2 and GPx to the β actin. The data are presented as mean ± SD Notes: #*P* < 0.05 (analysed by one-way ANOVA, post-hoc LSD test). (A) Magnification: 400×; Scale bars 100 μm

**Figure 3:**
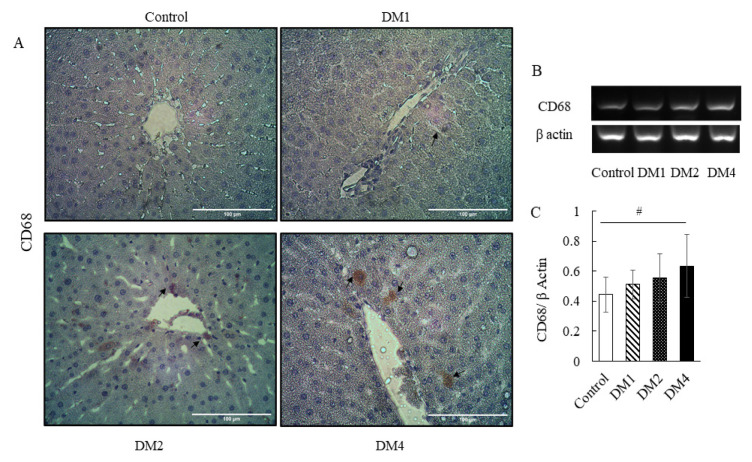
Immunohistochemical staining and mRNA expression of the macrophage marker CD68 in the rat liver. (A) Representative images of CD68 immunohistochemical staining. CD68-positive cells were found in periportal areas in the DM groups. The number of CD68-positive cells increased gradually followed the duration of DM, respectively (black arrows). (B) Representative images of gel electrophoresis from RT-PCR products of CD68 and β actin. (C) Relative quantification of the mRNA expressions of CD68 by densitometry analysis. The expression was normalised to the expression of a housekeeping gene β actin. CD68 mRNA expression was significantly upregulated in the DM4 group. The data are presented as mean ± SD Notes: #*P* < 0.05 (analysed by one-way ANOVA, post-hoc LSD test). (A) Magnification: 400×; Scale bars 100 μm

The liver of the control group revealed the normal histological structure of hepatic lobules. No histopathological alterations were observed (Figure 1). The DM groups showed a gradual alteration of liver morphology, including dilated and congested hepatic sinusoids, and hepatocytes with cytoplasmic vacuolisation. The liver morphological alteration was markedly observed in DM4 group (Figure 1). The liver cells in the DM1 group revealed a dark brown staining of SOD1, particularly in the cytoplasm (Figure 2A). In addition, the livers from the DM2 and DM4 groups showed a decrease of SOD1-positive cells compared to the DM1 and control groups (Figure 2A). The mRNA expressions of antioxidant enzymes (SOD1, SOD2 and GPx) in the DM1 group were slightly higher than the control group but failed to reach statistical significance (Figures 2B–2G). The mRNA expressions of SOD1 in the DM2 and DM4 groups were significantly lower than the control and DM1 groups (*P* < 0.05) (Figure 2B). The hepatic SOD2 mRNA expression in the DM4 group was significantly lower than the DM1 group (*P* < 0.05) (Figures 2C and 2F). The SOD2 and GPx mRNA expressions in the DM4 group were slightly lower than the control group, but statistically insignificant (Figures 2D and 2G). Diabetes induced an increase of CD68-positive cells in the livers of the DM groups (Figure 3A). The hepatic expression of CD68 was significantly increased in the DM4 group compared to the control group at the mRNA level (*P* < 0.05) (Figures 3B and 3C).

### Long-Term Diabetes Induced Increased Apoptosis and Senescence in the Rat Liver

**Figure 4:**
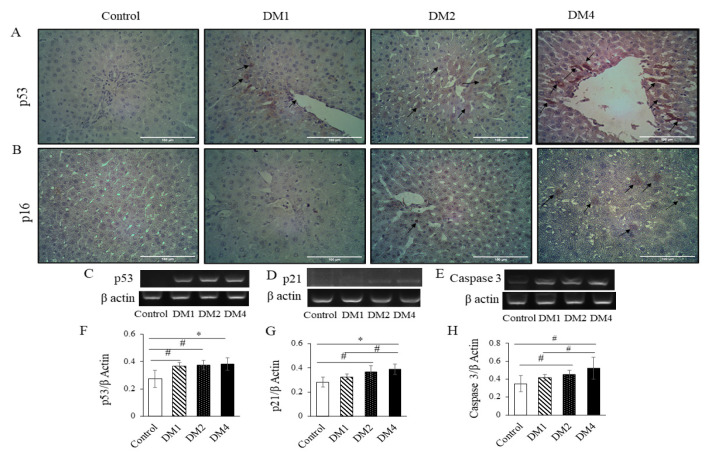
The p53 and p16 immunohistochemical staining and mRNA expressions of the p53, p21 and caspase-3 genes. (A) Representative images of p53 immunohistochemical staining. The black arrows indicate p53-positive cells in the DM groups. (B) Representative images of p16 immunohistochemical staining. The black arrows indicate p16 positive cells. p16-positive cells markedly observed in the DM4 group. (C–E) Representative images of gel electrophoresis from RT-PCR products of p53, p21, caspase-3 and its corresponding β actin, respectively. (F–H) Relative quantification of the mRNA expressions of p53, p21 and caspase-3 to the β actin. The data are presented as mean ± SD Notes: #*P* < 0.05, *P < 0.001 (analysed by one-way ANOVA, post-hoc LSD test); (A), (B): Magnification: 400 ×; Scale bars 100 μm

The p53-positive cells were progressively elevated in the livers of the diabetes groups (Figure 4A). The hepatic p53 mRNA expression was significantly higher in the DM1 (*P* < 0.05), DM2 (*P* < 0.05) and DM4 groups (*P* < 0.001) than the control group (Figures 4C and 4F). The mRNA expression of senescence marker p21 was significantly higher in the DM2 (*P* < 0.05) and DM4 groups (*P* < 0.001) than the control group and significantly higher in the DM4 group compared to the DM1 group (*P* < 0.05) (Figures 4D and 4G). The expression of apoptotic marker caspase-3 was also significantly higher in the DM2 and DM4 groups (*P* < 0.05) than the control group, and the DM4 group had a markedly higher expression of caspase-3 than the DM1 group (*P* < 0.05), at the mRNA level (Figures 4E and 4H). The senescence marker p16-positive cells were most prominent in the livers of the DM4 group and only a few p16-positive cells were found in the livers of the DM2 group (Figure 4B).

### Correlations between the p53, p21 and caspase-3

There was a significant positive correlation between mRNA expression of p53 and p21 (*P* = 0.001 and *r* = 0.628), between p53 and caspase-3 (*P* = 0.022 and *r* = 0.487) and between p21 and caspase-3 (*P* = 0.003 and *r* = 0.610) (Table 2).

## Discussion

The development of liver injury in diabetic rats involves progressive histological changes, including an increase in the number of hepatocytes with vacuolated cytoplasm indicating lipid droplet accumulation and an extensive dilatation of blood sinusoids. High blood glucose level might activate several pathways, including the polyol pathway leading to growth factor and cytokine production, and induce mitogen-activated protein kinase (MAPK) and PK-C activation. Evidence from clinical and experimental studies have shown that increased oxidative stress plays a crucial role in the initiation of diabetes and the progression of its complications (23, 24). Cells have an antioxidant system as a defense mechanism against ROS, but this defense system can still be disrupted by ROS causing damage to nucleic acids, proteins and lipids. Hyperglycaemia during diabetes stimulates ROS production and reduction of the cellular antioxidant defense system (25, 26). Increased oxidative stress in DM involves several processes including: the generation of ROS due to glycosylation, glucose auto-oxidation, nonenzymatic glycation and changes in the activity of antioxidant defense systems.

The increase of non-trapped free radicals by antioxidant enzymes during periods of oxidative stress will cause endothelial dysfunction and induce formation of AGEs (27, 28). Endogenous antioxidant enzymes, including superoxide dismutase (SOD), glutathione peroxidase (GPx) and catalase (CAT) serve as the first-line of defense against free radicals and protect cells from ROS-induced cellular damage. In the present study, the expression of SOD1/cytosolic SOD/copper zinc (CuZn-SOD) in immunohistochemistry was gradually decreased following the long-term duration of diabetes in the DM2 and DM4 groups. This result was also confirmed by a decrease in SOD1 in the DM2 and DM4 groups at the gene expression level. A tendency to decrease manganese (Mn-SOD)/SOD2 and GPx was also observed in the DM4 group at the mRNA level, but the decrease was not statistically significant. These findings may indicate that with a longer duration of diabetes, the antioxidant enzyme SOD1 tended to decline at the gene and protein levels, suggesting that long-term hyperglycaemia induces downregulation of the main antioxidant enzyme SOD1. Further research is warranted to quantify the antioxidant enzymes at the activity levels. However, other studies also demonstrated a decrease of SOD activity in the heart and the liver of diabetic animal models (29, 30). Decreased SOD activity was also found in the serum of type II diabetic patients with microalbuminuria and in erythrocyte samples of both type I and type II diabetic patients (31, 32). In contrast, another study reported that high glucose induced increased mRNA expressions of SOD1, CAT and GPx in peripheral blood mononuclear cells of type I diabetic patients without microvascular complications and decreased expression of these enzymes was found in patients with nephropathy (33). The subject, the type of samples, the study design, diabetes duration and experimental conditions may account for these differences.

SOD is the main antioxidant enzyme that counteracts the highly reactive superoxide anion, then catalyses the conversion of these free radicals to the less reactive forms (hydrogen peroxide [H_2_O_2_]) and to oxygen (O_2_) (34). A slight increase in the gene expressions of SOD1 and the other antioxidant enzymes in the DM1 group may be a compensatory mechanism to cope with the damage caused by hyperglycaemia in hepatocytes. Previous clinical and experimental animal studies reported increased SOD activity at the onset of diabetes which subsequently decreased thereafter (32, 35, 36). However, the current study did not analyse the expression of SOD1 in the rat liver at the early onset of diabetes. The decrease in SOD activity is partly due to glucose auto-oxidation in diabetic patients, resulting in the accumulation of H_2_O_2_, further triggering SOD inactivation (32, 37).

This study also demonstrated an increased expression of the inflammatory marker CD68 in the rat livers along with the increase of diabetes duration, suggesting involvement of macrophage cell lineage recruitment in the diabetic livers. The influx of macrophages activates the inflammatory pathway, mediates podocyte apoptosis, then progresses into diabetic nephropathy via the ROS-p38 MAPK signaling pathway (38). High blood glucose levels, the formation of AGEs and oxidised low density lipoproteins lead into macrophage accumulation and activation through induction of adhesion molecules and chemokines in the diabetic tissues (39). These findings suggest that increased macrophage infiltration plays an important role in the activation of inflammatory pathways during the progression of diabetes-related liver injuries.

High blood glucose induces ROS generation in various ways, which activates inflammatory pathways, then leading to the development of diabetic nephropathy (3). Therefore, prolonged hyperglycemic conditions might induce increased oxidative stress and inflammation, leading to severe diabetic conditions along with organ dysfunction. In addition, it has been described that chronic inflammation associated with increased oxidative stress, as well as altered nitric oxide signaling, is the main pathological condition of renal and cardiovascular disease in diabetic conditions (40, 41). In addition, increased oxidative stress and inflammatory cytokines stimulate an increased rate of apoptosis in diabetic nephropathy (42).

The long-term effects of diabetes along with a decrease in antioxidant defenses lead to an increase in liver cell apoptosis. The present study demonstrated increased mRNA expressions of apoptotic markers p53 and caspase-3 in the rat livers following the duration of hyperglycaemic condition of diabetes. High blood glucose induced apoptosis, at least in part, through the upregulation of p53 level and the activation of caspase-3 activity in the rat livers. Hyperglycaemia induces the increased activity of caspase-3 which is an important mediator of apoptosis in diabetic cardiomyopathy (3). The activated caspase-3, which is one of the downstream effector caspases, is the main trigger of apoptosis leading to cell death. The activation of apoptosis pathways can be triggered by the overproduction of mitochondrial ROS-induced by hyperglycaemia. Apoptosis or cell death with specific morphological features is a process that usually occurs during development, aging, and also as the body’s response to immune reactions or disease (43, 44). Oxidative stress causes loss of cell function, then cells undergo apoptosis or necrosis (45). Hyperglycaemia-induced oxidative stress is related to cellular death in the heart, retina, kidneys and nerves (24, 46).

ROS induces DNA damage and activates a response to such damage, further leading to activation of p53 (47). Chronic hyperglycaemia followed by an increase in cytoplasmic glucose concentration leads to C-terminal glycosylation of the tumor suppressor p53, then induces activation of the transcriptional activator p53 leading to its translocation to the nucleus, in which transcription of a number of p53-dependent genes is initiated (46, 48). The general response of various cell types that are exposed to DNA-damaging agents is usually characterised by upregulation of the p53 protein (49). The tumour suppressor gene p53 exhibits its pivotal role in response to DNA damage at the G1/S checkpoint, controls cell cycle progression, stimulates apoptosis and is also involved in the development of diabetic complications (46, 49). Hepatic p53 level was increased in type II diabetic patients and positively related to the insulin resistance (50).

Under stress conditions, p53 selectively regulates the expression of its target genes leading to cell cycle arrest, DNA repair, apoptosis or senescence (47, 50). The p53-upregulated modulator of apoptosis (PUMA) and phorbol-12-myristate-13-acetate-induced protein 1 (PMAIP1/Noxa) are target genes of p53 in apoptosis pathway, while cyclin dependent kinase inhibitor CDKN1A (p21) is a downstream target of p53 in cell cycle arrest or senescence (51). In response to mild stress, the activated p53 induces the growth-arrest gene p21 as a mediator of DNA damage repair; if DNA damage persists, p53 will trigger programmed cell death (apoptosis) (51). The p53 tends to activate cell cycle arrest and repair pathways in the low-grades of DNA damage, whereas p53 induces pro-apoptotic genes, caspase activation leading to apoptosis or cell death in the high-grades of DNA damage (52, 53).

In vitro studies reported that hyperglycaemic conditions induce activation of p53 and its downstream mediators leading to myocyte apoptosis (46). In the present study, hyperglycaemic conditions might induce ROS generation and inflammation leading to p53 activation, then p53 mediated cellular senescence and apoptosis in the diabetic livers. Therefore, p53 controls cell-fate determination and regulates several processes including cell cycle arrest, senescence and apoptosis in the livers of the diabetic rats. These findings suggest that prolonged hyperglycaemia induces p53-mediated senescence and apoptosis, which then leads to the development of liver injury in diabetic conditions.

This study demonstrated a positive correlation between mRNA expression of p21 and caspase-3 in the liver of diabetic rats. Although it has been known that p21 protects the DNA-damaged cells from apoptosis, different levels of stress condition and cell type might contribute to this discrepancy (53). Further studies are needed to confirm these results. Whether p53/p21-pathway also enhances apoptosis is not clearly understood. A previous study reported that p21 can act either in an antiapoptotic or a proapoptotic role (54). It has also been demonstrated that p21 promotes apoptosis via the intrinsic apoptotic pathway of mitochondria in the β cells (55). In addition, another study reported that p21 can potentiate the effect of bile acids in inducing hepatocyte apoptosis (54).

The present study revealed that hyperglycaemia induced cellular senescence not only via upregulation of p53/p21 signaling, but also via activation p16INK4a (p16), as characterised by increased expression of the p16-positive cells in the diabetic livers. The activation of p16 is mediated by activated MAPK due to ROS induction (56). The p53/p21 signaling pathway plays an important role in responding to DNA damage as well as p16, which is a cyclin-dependent kinase inhibitor, another major mediator in cellular senescence (47). The p16 plays a pivotal role in G1 cell-cycle arrest by activation of the retinoblastoma tumor suppressor protein (pRb) in cellular senescence. The p53/p21 pathway activates cell cycle arrest in the early stages of senescence or initiates the senescence, while p16 plays a role in maintaining a state of senescence (56, 57). In the present study, the expression of p16-positive cells was markedly increased in the DM4 group compared with the DM2 group. These findings indicate that liver cells respond to hyperglycaemic conditions via p16-mediated cell senescence at a relatively late stage. It has been suggested that senescence once demonstrated by the p16/Rb signaling pathway is an irreversible state (58–60). Senescent cells may cause disruption of normal tissue structure and reduce its function (60). Taken together, hyperglycaemia conditions may result in increasing oxidative stress, enhancing DNA damage; then it induces upregulation of p53-mediated both senescence and apoptosis, activation of p21, coupled with p16 activation of senescence. Hyperglycaemia might induce premature senescence in liver cells by activating the p53/p21 and p16 pathways. These events, then inhibit cell proliferation, accelerate aging and might reduce liver function. We realised the limitations of our study that measurements of oxidative stress biomarkers such as the assessment of oxidative damage, including malondialdehyde, protein carbonyl, have not been identified quantitatively. Therefore, we were not able to determine the level of oxidative stress. Further studies are needed to establish measurements of these markers or other approaches to quantify the level of oxidative stress in the diabetic liver.

## Conclusion

In summary, hyperglycaemia is responsible for apoptotic cell death, premature senescence and pathological alterations in diabetic rat liver. Importantly, a strong association between p53 upregulation with both apoptosis and senescence has been identified in the diabetic liver. The upregulation of hepatic p53 might contribute to the progression of liver injury in DM.

## Figures and Tables

**Table 1:** List of primers used in this study

No.	Gene	Sequences	Annealing temperature (°C)
1	Cluster of differentiation 68 (CD68)	forward 5’-TGTGTCCTTCCCACAAGCAG-3’ reverse 5’-AAGAGAAGCATGGCCCGAAG-3’	60
2	p53	forward 5’-CCCCTGAAGACTGGATAACTGT-3’ reverse 5’-ATTAGGTGACCCTGTCGCTG-3’	52
3	p21	forward 5’-GTGATATGTACCAGCCACAGG-3’ reverse 5’-CAGACGTAGTTGCCCTCCAG-3’	55
4	Caspase-3	forward 5’-CCGACTTCCTCTATGCTTACTC-3’ reverse 5’-CGTACAGTTTCAGCATGGC-3’	57
5	SOD1	forward 5’-GCGGTGAACCAGTTGTGGTG-3’ reverse 5’-AGCCACATTGCCCAGGTCTC-3’	55
6	SOD2	forward 5’-ATGTTGTGTCGGGCGGCGTGCAGC-3’ reverse 5’-GCGCCTCGTGGTACTTCTCCTCGGTG-3’	58
7	GPx	forward 5’-CTCTCCGCGGTGGCACAGT-3’ reverse 5’-CCACCACCGGGTCGGACATAC-3’	59
8	An internal control gene β actin	forward 5’-GCAGATGTGGATCAGCAAGC-3’ reverse 5’-GGTGTAAAACGCAGCTCAGTAA-3’.	54

**Table 2:** Association analyses of p53, p21 and caspase-3

	p53	p21	caspase-3
p53	-	0.628/0.001#	0.487/0.022#
p21	-	-	0.610/0.003#

Notes: The data are presented as the *r*-value/*P*-value. The *r*-value indicates the Pearson’s correlation coefficient;

# *P* < 0.05
